# Supplementary material for: Shared Treg dysregulation underlies psoriasis vulgaris and atopic dermatitis through the PHF19-PRC2 epigenetic axis
Source: Front Immunol. 2026 Aug 20;17:1932788. doi: 10.3389/fimmu.2026.1932788 (PMC13538097; doi:10.3389/fimmu.2026.1932788)
Supplement: Supplementary file 3 [file DataSheet3.zip › Additional files/Additional file 1/40.RPL8.scatter.pdf]

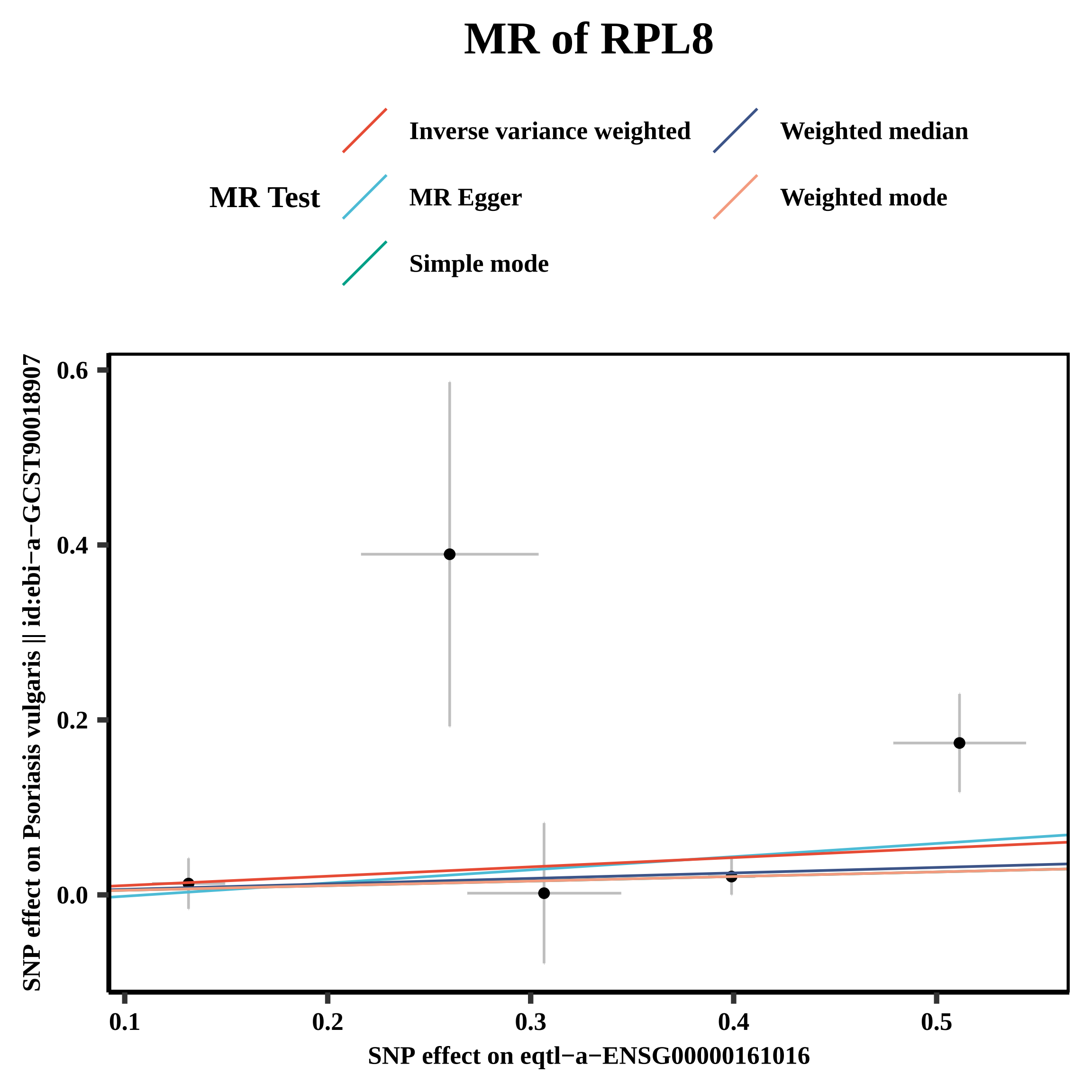

MR of RPL8
MR Test
Inverse variance weighted
Weighted median
MR Egger
Weighted mode
Simple mode
0.6
0.4
0.2
0.0
0.1
0.2
0.3
0.4
0.5
SNP effect on Psoriasis vulgaris || id:ebi−a−GCST90018907
SNP effect on eqtl−a−ENSG00000161016
